# Effects of neuromuscular versus plyometric training on physical fitness and mental well-being in male pubertal soccer players

**DOI:** 10.1038/s41598-025-30142-x

**Published:** 2025-12-08

**Authors:** Achraf Hammami, Abdelkader Mahmoudi, Walid Selmi, Yassine Negra, Haithem Rebai, Urs Granacher, Raouf Hammami

**Affiliations:** 1 Higher Institute of Sport and Physical Education of Ksar-Said, University of Manouba, University Campus, Manouba, 2010 Tunisia; 2 Tunisian Research Laboratory Sports Performance Optimization, National Center of Medicine and Science in Sports (CNMSS), Tunis, Tunisia; 3 Research Laboratory (LR23JS01) Sport Performance, Health and Society, Tunis, Tunisia; 4 Department of Sport and Sport Science Exercise and Human Movement Science, University of Freiburg, Sandfangweg 4, 79102 Freiburg, Germany

**Keywords:** Athletic performance, Behavior, Football, Psychological state, Youth athletes, Health care, Neuroscience, Physiology, Psychology, Psychology

## Abstract

Neuromuscular training (NMT) and plyometric training (PT) are commonly used during long-term athlete development, yet their relative effects on physical fitness and mental well-being are not fully understood. This study compared 8 weeks of NMT versus PT on physical fitness, mental well-being, emotional intelligence, and attention in pubertal male soccer players and explored associations between training-induced changes in these domains. Twenty-four male soccer players (12.3–12.5 years, circa-peak height velocity: − 0.7 to − 0.8) were randomly assigned to NMT or PT. Both groups trained twice weekly in addition to regular soccer practice. NMT included balance, strength, plyometric, change-of-direction, and agility exercises, while PT focused on bilateral and unilateral jump-landing drills. Training volumes were matched. Physical fitness tests included the five-time jump test, 20-m sprint, and 15-m change-of-direction speed test. Mental well-being outcomes included cognitive and somatic anxiety, self-confidence, attention, and emotional intelligence. PT led to larger improvements in jump, sprint, and change-of-direction speed performances, whereas NMT produced greater gains in self-confidence, anxiety regulation, attention, and emotional intelligence. A graphical summary illustrates the distinct physical and psychological adaptations to PT and NMT, highlighting their complementary nature. The observed fitness improvements significantly correlated with changes in psychological outcomes. These findings suggest that strength and conditioning professionals should prioritize PT when aiming to enhance physical fitness and NMT when targeting psychological well-being, supporting a holistic approach to athletic development in pubertal soccer players.

## Introduction

In youth soccer, adequate levels of physical fitness (e.g., muscular strength, power, and change-of-direction speed [CoD])^1^ and balanced mental well-being (including self-esteem, life satisfaction, and overall happiness)^2^ are essential to meet the general and specific demands of training and competitive match play. Accordingly, fostering physical fitness and mental well-being from an early age on is important to enable young players to engage in their sport in a physically and mentally healthy manner^3^. There is evidence that neuromuscular training (NMT) and plyometric training (PT) are both effective to improve soccer-related physical fitness and mental well-being^4,5^.

While PT is a highly specific training type that focuses on exercises performed in the stretch–shortening cycle (SSC)^6^, NMT is a multimodal regime including balance, muscle strength, power and speed exercises. A meta-analysis of Ramirez-Campillo, et al.^7^ included 57 studies and indicated that PT has the potential to induce significant and large effects on measures of physical fitness (e.g., jump performance, linear sprint and CoD speed) and sport-specific performance in male and female young athletes. With regards to mental well-being, Znazen, et al.^8^ investigated the effects of three different PT programs conducted at low, moderate, or high intensities on attention and mood states in young adults aged 19 years. The authors reported no significant changes in mood sub-scales across the three different PT programs.

Regarding NMT, Williams, et al.^9^ conducted a systematic review and meta-analysis of nine studies examining its effects on physical fitness in healthy youth male and female athletes aged 8–18 years. Findings revealed training-induced improvements in muscle strength, power, speed, and balance. Moderator analyses according to age and sex showed that NMT resulted in larger effects in younger (under 13.8 years) compared to older participants (over 13.8 years), and in male compared to female individuals^9^. Moreover, Hammami, et al.^10^ recently reported that 8 weeks of NMT not only improved physical fitness (balance, CoD speed and muscle power) but also positively affected mental well-being, such as cognitive, somatic anxiety, and self-confidence in pubertal male soccer players aged 12.2 ± 0.6. In another study, Duncan, et al.^11^ showed that 10 weeks of NMT implemented in physical education enhanced self-esteem to a greater extent than regular physical education in boys aged 6 to 7 years.

These findings underscore the importance of integrating NMT to foster an integrated approach focussing on physical and psychological development in pubertal athletes. The observed findings are likely due to the varied cognitive demands attributed to NMT exercises, which may enhance self-efficacy and emotional regulation^11^. Hence, multimodal NMT may offer a broader spectrum of adaptations compared with single-mode PT in youth athletes. While PT research has predominantly focused on improving physical fitness such as proxies of muscle power and speed^7^, limited attention has been given to its effects on psychological dimensions. In contrast, NMT, which has the potential to enhance strength, speed, agility, and proxies of muscle power^4^, may offer additional benefits by fostering mental well-being and emotional intelligence^11^. To our knowledge, there is no study available that has directly contrasted the physical and psychological adaptations elicited by PT and NMT in youth athletes. Finally, such research addresses a key gap in youth sport by examining physical fitness and psychological adaptations to training during puberty. Although adolescence is a critical period for neuromuscular and emotional development^12–14^, few studies have examined how training interventions can simultaneously target both dimensions.

Therefore, the aim of this study was to examine the effects of NMT versus PT on selected components of physical fitness (muscle power, linear sprint and CoD speed) as well as mental well-being (e.g., somatic and cognitive anxiety, self-confidence), emotional intelligence (total intra and inter emotional intelligence) and attention in male pubertal soccer players. A secondary aim was to investigate associations between training-induced changes in physical fitness and mental well-being, emotional intelligence, as well as attention to examine whether improvements in one domain are related to improvements in the other domain. In accordance with the established principle of training specificity^15^, we hypothesized that PT results in larger fitness improvements than NMT in trained pubertal soccer players^7,16,17^. With regards to mental well-being, emotional intelligence and attention, there is limited evidence indicating that NMT might cause superior effects compared with PT in youth^10,11^.

### Study design

This study is a randomized controlled trial that set out to examine the effects of NMT versus PT on physical fitness and mental well-being in male pubertal soccer players. The independent variables are the different training modalities (NMT, PT). In addition, the main dependent variables comprise soccer-related physical fitness tests (e.g., jump, linear sprint and CoD speed tests) as well as mental well-being (e.g., somatic and cognitive anxiety, self-confidence), emotional intelligence (total intra and inter emotional intelligence) and attention. The fitness tests were applied pre and post the 8-week intervention program. Participants were randomly assigned to the NMT or PT. Both training groups participated in an 8-week training program with two sessions per week in addition to the regular soccer training that focused on the promotion of soccer-specific technical drills. Training volumes were similar between groups.

A 15-min dynamic warm-up was performed before each training session, including low-intensity jogging, dynamic stretching, forward, sideways, and backward running, acceleration runs, and submaximal vertical and horizontal jumps. Participants also engaged in regular school-based physical activity, averaging 3 h per week, which contributed to their overall weekly exercise volume.

## Methods

### Participants

The sample size estimation was computed using G*Power software (version 3.1.6). Based on findings from a related study from, Hammami, et al.^18^ who examined the effects of NMT on 15-m CoD speed (Cohen’s f = 0.48) in highly-trained male youth soccer players (Tier 3), an a priori power analysis with a type I error of 0.01 and 90% statistical power was computed. The analysis indicated that 20 participants would represent a sufficient sample. For the purpose of this study, we recruited a total of 24 trained male pubertal soccer players from the same team. These players were randomly assigned to either a NMT (n = 13) or a PT group (n = 11) (Table 1). All participants had 4 ± 0.2 years of organized soccer experience and played in various positions, including defenders, midfielders, and forwards, ensuring a comparable skill and positional distribution across groups. All physical tests were conducted on a third-generation synthetic soccer turf at the soccer academy of Takelsa, Nabeul, Tunisia, under standardized conditions.

**Table 1:** Anthropometrics of the examined study cohort according to group allocation.

	NMT (n = 13)	PT (n = 11)	*p*-value
Age (years)	12.3 ± 0.6	12.5 ± 0.3	0.745
Body height (cm)	156.0 ± 6.4	160.4 ± 9.0	0.841
Body mass (kg)	50.7 ± 4.6	53.8 ± 7.6	0.941
Maturity offset (years)	−0.7 ± 0.6	−0.8 ± 0.3	0.862
Predicted APHV (years)	13 ± 1.2	13.3 ± 0.6	0.756

Data are presented as means and standard deviations; NMT: neuromuscular training group; PT: plyometric training group.

Following McKay, et al’s .^19^ classification of athletes’ training and performance calibre, our participants can be categorized as Tier 2 (trained/developmental) athletes, with at least 4 years of systematic soccer training experience. It is important to note that both experimental groups followed the identical regular soccer training program, supervised by the same coaches. The NMT and PT groups specifically incorporated two weekly PT or NMT training sessions (Table 2). To account for individual development, each participant’s biological maturity status was estimated using the maturity offset method, based on the prediction equation of Moore, et al.^20^.

**Table 2:** Exemplified micro-cycle of the neuromuscular training and plyometric training programs in combination with the soccer-specific training.

Week days	Monday	Tuesday	Wednesday	Thursday	Friday	Saturday	Sunday
Training type							
Warm-up	Day off	Submaximal running, dynamic stretching acceleration and deceleration drills, and jump – landing tasks (15 min)					Competition day
Intervention		NMT or PT (30 min)	No NMT or PT	NMT or PT (30 min)	No NMT or PT	No NMT or PT	
			Technical and tactical drills (35 min)		Technical and tactical drills (35 min)	Technical and tactical drills (35 min)	
			Small-sided games with or without goal (35 min)		Small-sided games with or without goal (35 min)	Small-sided games with or without goal (35 min)	
Soccer-specific training		Technical and tactical drills (20 min)		Technical and tactical drills (20 min)			
		Small-sided games with or without goal (20 min)		Small-sided games with or without goal (20 min)			
Cool down		Jogging and dynamic stretching (5 min)					
Total session volume		90 min	90 min	90 min	90 min	90 min	

Technical drills: ball control, ball pass and dribbling, feints and moves, positional rotations, combination passing, crossing, ball kicking etc. Tactical drills: Soccer-specific defending drills for specific defending positions such as centre defenders, outside backs etc.

Soccer-specific attacking drills for specific attacking positions such as centre forwards, wingers, attacking mid-fielders etc. NMT: neuromuscular training; PT: plyometric training.

Before study participation, players and their legal representatives received information on the study procedures and goals, potential risks, and benefits. Informed consent was obtained from both, the legal representatives and the players. This study adhered to the latest version of the Declaration of Helsinki, and the protocol received approval from the Local Ethics Committee of the National Centre of Medicine and Science of Sports of Tunis (CNMSS-LR09SEP01) prior to study commencement. None of the participating players suffered before (6 months) and during the study from psychological, musculoskeletal, neurological, or orthopedic disorders.

### Procedures

A week before the study began, all players attended a familiarization session to become accustomed with the fitness tests and PT as well as NMT exercises. Players assigned to NMT and PT received specific instructions on proper exercise techniques. All participants completed the physical fitness and psychological assessments in a fixed order across sessions. Standardized rest periods were provided between tests to minimize fatigue and ensure consistent testing conditions. The same test sequence was used during pre- and post-testing. Test instructors were unaware of group allocation. To minimize potential fatigue effects on cognitive performance, athletes first completed the five-jump test (FJT), followed by the 15-m CoD and the 20-m linear sprint speed tests, with standardized rest intervals between trials. The mental well-being, emotional intelligence, and attention assessments were then conducted after ensuring adequate recovery time.

Before the physical fitness tests started, all participants conducted a standardized 10-min warm-up including balance exercises (forward/backward beam walking and single-leg stances on unstable devices), submaximal running drills (skipping), and landing drills (snap downs and single-leg drop squats). A 5 min rest period separated each test, with a 3-min rest between individual test trials^21^. For the physical fitness tests (FJT, CoD, linear sprint), the best out of two trials was recorded for statistical analysis. For the cognitive, somatic anxiety, self-confidence, attention and emotional intelligence assessments, only one trial was performed. All attention and emotional intelligence tests were administered individually in quiet, controlled rooms, under the supervision of trained researchers, to ensure standardized conditions and minimize potential distractions.

A passive control group was not included in this study because it is unethical to not allow young athletes to train for a certain period of time^22,23^. Since authors from previous studies have already shown that NMT is generally effective for fitness enhancement in young pubertal soccer players^10,18^, our main goal was to directly compare the specific effects of NMT versus PT.

### Anthropometrics

Body height was measured using a wall-mounted stadiometer (Florham Park, NJ) and body mass with an electronic scale (Baty International, West Sussex, England). To estimate body composition, we measured the sum of skinfolds using Harpenden’s skinfold calipers. Subsequently, we non-invasively assessed biological maturity using the maturity offset method according to Moore, et al.^20^, which has demonstrated consistent prediction errors across both adult and adolescent populations. Accordingly, athlete’s chronological age and body height were included in the following regression equation:

Maturity offset = 27.999994 + (0.0036124 × age × height)^20^.

### Physical fitness tests

#### Proxies of muscle power

The FJT was used as a proxy to estimate muscle power, following the guidelines of Chamari, et al.^24^. Players started the test in standing position with both feet flat on the ground and performed five alternating left and right leg bounds, aiming to cover the maximum possible horizontal distance. As dependent variable, the horizontal jump distance was tested to the nearest centimeter using a tape measure. This test has previously shown high test–retest reliability, with an ICC of 0.91 for youth soccer players^25^.

#### Change-of-direction (CoD) speed

CoD speed was measured using the 15-m CoD test. Athletes began with a 3-m linear sprint before entering a 3-m slalom section marked by three 16-cm high pylons, spaced 1.5 m apart. After navigating the pylons, athletes cleared a 0.5-m hurdle positioned just beyond the final pylon^26^. As dependent variable, the best time out of two trials was to taken to complete the test. The 15-m CoD test has demonstrated excellent test–retest reliability, with an ICC value of 0.93^26^.

#### Linear sprint speed

For the 20-m linear sprint speed test, players sprinted as fast as possible from a starting line. The sprint time was automatically recorded using photocell gates (Brower Timing Systems, Salt Lake City, UT, USA; accuracy of 0.01 s) positioned 0.4 m above the ground. Each player completed two trials and rested for 5 min between trials. The best (shortest) time was used for further analysis. This test has previously demonstrated excellent test–retest reliability in young soccer players (ICC = 0.97)^27^.

### Mental well-being tests

#### Tests for the assessment of anxiety and self-confidence

Participants’ competitive state anxiety was tested using the Competitive State Anxiety Inventory-2 (CSAI-2). The Arabic translation of the questionnaire, validated with 13 items by Boudhiba, et al.^28^ was applied. The CSAI-2 is a widely recognized tool for assessing multi-dimensional anxiety in athletes within competitive environments. This inventory evaluates three core components. First, cognitive anxiety which reflects worries and negative thoughts about performance (e.g., “I am concerned about this competition,” “I am concerned about choking under pressure”); second, somatic anxiety that pertains to the physical symptoms of anxiety, such as increased heart rate or muscle tension (e.g., “I feel nervous,” “I feel tense in my stomach”); and third, self-confidence which represents an athlete’s belief in his ability to perform successfully (e.g., “I feel at ease,” “I am confident I can meet the challenge”).

Participants responded to each item on a 4-point Likert scale, indicating “how do you feel right now” from “not at all” to “very much so.” Each of the three subscales (cognitive anxiety, somatic anxiety, and self-confidence) consists of 13 items. The scores for these items were summed to provide an intensity level for each component. This tool offers valuable insights into how these psychological factors interact with physical fitness, providing a robust framework for tailoring interventions to reduce anxiety and boost self-confidence in young athletes. The translated CSAI-2 included 13 items and it has previously demonstrated excellent test–retest reliability in youth athletes with ICC values of 0.94 for cognitive anxiety (CA), 0.87 for somatic anxiety (SA), and 0.79 for self-confidence (SC), respectively^29^.

#### Tests for the assessment of emotional intelligence (EI)

Emotional intelligence (EI) was assessed using the Psychometric Emotional Competence (PEC) scale. Participants responded to 50 items on a 5-point Likert scale (1: strongly disagree, 2: disagree, 3: neutral, 4: agree and 5: strongly agree). The PEC measures both intrapersonal emotional competence (understanding one’s own emotions) and interpersonal emotional competence (understanding others’ emotions) as separate constructs. The instrument also provides a global score representing overall emotional competence. The PEC has previously demonstrated excellent reliability with ICC values ranging between 0.90 and 0.98 for all of the studied items^30^.

#### Tests for the assessment of attention

The d2 test was used to evaluate participants’ selective attention, concentration, and mental speed, and is widely recognized for its reliability and validity. The test demonstrates excellent reliability, with ICCs ranging from 0.95 to 0.98 across variables^27^, and strong criterion, construct, and predictive validity^28^. The test consists of 14 lines, each containing 47 letters, including the target letters “p” and “d” with 1–4 small marks. Participants were instructed to quickly scan each line and cross out every “d” with exactly two marks, while ignoring all other letters and symbols. Each line was completed within 20 s. The dependent variable was the total d2 test score, calculated as the number of correctly identified target letters minus the number of errors, reflecting attention and concentration performance.

### Training programs

Both training programs lasted 8 weeks and were integrated into the players’ regular in-season soccer training from February to March 2025. Prior to the intervention, all participants followed a typical in-season routine consisting of five weekly soccer training sessions (Tuesday–Saturday), with Sunday designated for competition and Monday for recovery. None of the players had previously participated in NMT or PT programs, ensuring that both training modalities represented novel exercise stimuli for all participants. Each 90-min session began with a standardized 15-min dynamic warm-up (including dynamic stretching, submaximal running, acceleration and deceleration drills, and jump–landing tasks). On Tuesday and Thursday, 30-min blocks of NMT or PT replaced an equivalent portion of the regular soccer-specific training. After these sessions, players performed 40 min of soccer-specific drills, consisting of 20 min of technical and tactical exercises and 20 min of small-sided games with or without goals. On Wednesday, Friday, and Saturday, players completed 70 min of soccer-specific drills following the warm-up (35 min of technical/tactical work and 35 min of small-sided games). All sessions concluded with a 5-min cool-down (Table 2).

NMT primarily included five exercises designed to improve balance, strength/power, linear sprint and CoD speed as well as agility. Players performed 3 sets of 5–10 repetitions for each exercise, with a rest of 60–120 s between sets and exercises^31^. To ensure progressive overload, the rate of perceived exertion (RPE) was adjusted every two weeks using a 0–10 OMNI scale. During weeks 1–2, we targeted an RPE score of 3. During weeks 3–4, an RPE score of 5–6 was programmed, and during week 8, the RPE ranged between 7 and 8. All NMT exercises were conducted directly on the soccer pitch (Table 3).

**Table 3:** Design of the eight weeks neuromuscular training program.

Exercises	Weeks 1–2	Weeks 3–4	Weeks 5–6	Weeks 7–8
Balance	Single leg stance balance 3 sets of 5 repetitions	Single leg stance balance then controlling the ball around the body with the other leg 3 sets of 5 repetitions	Single leg balance stance: 3 sets of 5 repetitions	Acceleration of 2-m and vertical jumping before standing on single leg stance passing the ball with the dominant leg, then non-dominant leg 3 sets of 5 repetitions
Strength	Body mass half squat and body mass single leg squat 2 sets of 10 reps Nordic hamstring exercises 1 set of 5 repetitions	Half squat and side lunges with a 2 kg medicine ball 3 sets of 8 repetitions Nordic hamstring exercises 2 sets of 5 reps	Bulgarian split squats and 4 direction lunges with a 2 kg medicine ball 3 sets of 10 reps Nordic hamstring exercises 2 sets of 8 repetitions	Bulgarian squats and 4 direction lunges with a 2 kg medicine ball 3 sets of 10 reps Nordic hamstring exercises 2 sets of 8 repetitions
Power (i.e., plyometrics)	Standing long jump 2 sets of 10 repetitions	Side to side ankle hops over the hurdle 3 sets of 8 repetitions	Double and one-legged lateral jumps over the hurdle 3 sets of 10 repetitions	Cone hops with 180^◦^ side-cuts 3 sets of 10 repetitions
Agility	A pre-planned 3-m lateral shuttle run performed to the right and left (or vice versa), emphasizing quick directional changes and control. Upon completing the movement sequence, the player reacts to a visual cue (e.g., coach’s signal) to execute an accurate ball pass, combining reactive decision-making with speed and technical precision	Dribbling with multiple directional changes within a 4-m square while reacting to a visual cue (e.g., coach’s signal). The player must control and stop four balls at designated points, covering a total distance of 18 m, emphasizing coordination, reaction speed, and precise ball control under dynamic conditions	Hexagonal setup (2 m side length), where the player moves in a pre-planned sequence to touch cones numbered 1 to 4 before completing the drill with a ball pass. The total movement distance was 20 m, emphasizing quick directional changes, spatial awareness, and controlled execution	The player performs a 2-m acceleration while dribbling, weaves through three cones in a slalom pattern, executes a pass, and then immediately accelerates 5 m without the ball in response to a visual cue (e.g., the coach’s command). The total distance covered is 10 m, emphasizing agility, ball control, and reactive speed

At the end of each training week, coaches tested whether athletes were able to perform two more repetitions per set. Once this was achieved, the intensity or difficulty/complexity of the exercises was enhanced during neuromuscular training.

The PT program comprised bilateral and unilateral jump-landing exercises performed in vertical, horizontal, and lateral directions, emphasizing the SSC. The general structure and progression of the plyometric exercises were adapted from Bogdanis et al. (2019), who examined the effects of bilateral and unilateral PT on physical fitness. While the current study drew on that framework for plyometric exercise selection, the overall training design was expanded to specifically target performance components relevant to youth soccer namely, strength, muscle power, sprinting, CoD speed and agility. The intensity, volume, and progression of the exercises were prescribed following established PT guidelines for youth athletes^37^, ensuring an appropriate load to induce neuromuscular adaptations while minimizing injury risk. Each session included exercises performed in sets and repetitions consistent with prior youth PT interventions, with progression over the 8-week period to maintain adequate training stimuli. More specifically, exercises included drop jumps from a 20-cm drop height, horizontal jumps and lateral hops. Each session involved 3–4 sets of 6–12 repetitions for the three different plyometric exercises (Table 4). Progression was ensured by increasing foot contacts and varying exercise complexity. Participants were instructed to perform all exercises in the SSC at maximal effort and with minimal ground contact time.

**Table 4:** Design of the eight weeks plyometric training.

Weeks/Exercise	Week 1	Week 2	Week 3	Week 4	Week 5	Week 6	Week 7	Week 8
Drop jumps from 20-cm drop height	3 × 6	3 × 8	4 × 8	4 × 10				
Horizontal jumps	3 × 6	3 × 8	4 × 8	4 × 10				
Lateral hops	3 × 6	3 × 8	4 × 8	4 × 10				
Unilateral drop jumps from 20-cm drop height					3 × 3 each leg	3 × 4 each leg	4 × 4 each leg	4 × 5 each leg
Unilateral horizontal jumps					3 × 3 each leg	3 × 4 each leg	4 × 4 each leg	4 × 5 each leg
Unilateral lateral hops					3 × 3 each leg	3 × 4 each leg	4 × 4 each leg	4 × 5 each leg
Total foot contacts	54	72	96	120	54	72	96	120

Total training volume and intensity (overall time and effort spent training) were similar between PT and NMT. Training was supervised by qualified coaches and experienced sport scientists to ensure safety and effectiveness throughout the study period.

### Statistical analyses

Data are presented as group mean values and standard deviations (SD). After data normality was confirmed using the Shapiro–Wilk test, a MANOVA was applied to detect baseline between-group differences. A 2 × 2 analysis of variance (ANOVA) with repeated measures was computed on the factors group (NMT, PT) and time (pre, post) to determine training effects. Post-hoc tests with Bonferroni adjustments were conducted to identify group-specific pre- to post changes. Effect sizes for main time and group effects as well as group-by-time interactions were taken from the ANOVA output (partial eta squared transferred to Cohen’s d). Within-group Cohen’s d effect sizes (ES) were also calculated using the equation: d = (mean post − mean pre-) / mean SD.

The effect size d can be classified as small (0.00 < d < 0.49), moderate (0.50 ≤ d < 0.80), and large (d > 0.80)^32^. Pearson’s correlation coefficients were computed to assess potential associations between selected measures of physical fitness and mental well-being in pubertal soccer players. Correlation coefficients were considered trivial (r < 0.1), small (0.1 < r < 0.3), moderate (0.3 < r < 0.5), large (0.5 < r < 0.7), very large (0.7 < r < 0.9), nearly perfect (0.9 < r < 1.0), and perfect (r = 1.0)^33^. The level of significance was established at p < 0.05 and SPSS 20.0 was used for statistical analyses (SPSS Inc., Chicago, IL, USA).

## Results

All participants received treatments as allocated. The adherence rates were 92 and 90% for NMT and PT participants, respectively. No significant between-group baseline differences were observed for any of the tested variables (Tables 1 and 5). No training or test-related injuries were recorded over the course of the study. Main effects of group, time, and group-by-time interactions are displayed in Table 6. Figure 11 provides an integrated summary of the main between-group differences and training-specific adaptations, illustrating the relative emphasis of each intervention on physical versus psychological outcomes.

**Table 5:** Group-specific means and standard deviations for all outcome measures before (pre) and after (post) the intervention periods.

	PT (n = 11)				NMT (n = 13)				ANOVA	
	Pre test		Post test		Pre test		Post test		p-value (d)	
	M	SD	M	SD	M	SD	M	SD	Time	Group × Time
Physical fitness tests										
FJT (m)	8.7	0.8	10.2	0.8	8.7	0.5	9.2	0.3	< 0.001 (4.1)	< 0.001 (1.99)
15-m-CoD speed (s)	3.5	0.2	3.1	0.2	3.5	0.1	3.4	0.2	< 0.001 (4.52)	< 0.001 (.07)
20-m linear sprint speed (s)	2.9	0.3	2.7	0.3	2.8	0.4	2.7	0.4	< 0.05(1.07)	> 0.05 (0.20)
Mental well-being tests										
Cognitive anxiety (%)	13.3	3.8	12.6	1.9	14.6	3.5	10.3	2.8	< 0.01 (1.36)	< 0.05 (0.96)
Somatic anxiety (%)	13.7	1.8	11.5	1.5	13.7	3.7	9.6	2.3	< 0.001 (3.07)	< 0.05 (0.96)
Self confidence (%)	25.9	3.9	28.1	3.8	25.3	5.6	33.6	1.8	< 0.001 (2.29)	< 0.01(1.3)
Emotional intelligence tests										
EC total (%)	173.5	3.2	178.2	2.2	173.2	1.9	189.6	4.1	< 0.001 (7.29)	< 0.0014.03
EC intra (%)	84.2	3.4	89.7	3.6	83.4	1.8	97.7	2.7	< 0.001 (9.12)	< 0.0014.13
EC inter (%)	84.5	2.9	89.7	2.8	86.8	2.5	99.2	5.5	< 0.001 (3.27)	< 0.01 (1.34)
Attention test										
d2 test score (total score*)	137.5	6.3	140.2	5.7	140.2	2.7	151.4	2.7	< 0.001 (7.1)	< 0.0014.29

NMT: neuromuscular training; PT: plyometric training; FJT: five jump test, EC total: total emotional intelligence; EC intra: intra-emotional intelligence; EC inter: inter-emotional intelligence; d = Cohen’s d. *The d2 test score is derived from the number of correctly identified letters, subtracting by any error made.

**Table 6:** Correlational analyses of pooled data from all two experimental groups between training induced pre-post changes (deltas ∆) in selected measures of physical fitness, mental well-being and emotional intelligence variables in youth male soccer players (whole sample, N = 24).

		∆ CA (%)	∆ SA (%)	∆ SC (%)	∆ EC total (%)	∆ EC intra (%)	∆ EC inter (%)	∆ d2 test score (total score)*
∆ FJT (%)	r	− 0.357	− 0.525**	0.295	0.633**	0.661**	0.404*	0.643**
	*p*	0.086	0.008	0.162	0.001	0.000	0.050	0.001
	N	24	24	24	24	24	24	24
∆15 − m CoD test (%)	r	0.403	0.556**	− 0.308	− 0.656**	− 0.707**	− 0.289	− .594**
	*p*	0.051	0.005	0.143	0.0001	0.0001	0.172	0.002
	N	24	24	24	24	24	24	24
∆ 20 − m sprint test (%)	r	0.292	0.370	0.417*	0.012	0.134	− 0.087	0.315
	*p*	0.166	0.075	0.042	0.954	0.533	0.687	0.134
	N	24	24	24	24	24	24	24

FJT: five jump test; CoD: change-of-direction; CA: cognitive anxiety; SA: somatic anxiety; SC: self-confidence; EC total: total emotional competence; EC intra: intra emotional competence; EC inter: inter emotional competence. *The d2 test score is derived from the number of correctly identified letters, subtracting by any error made.

### Physical fitness tests

#### Proxies of muscle power

**Fig. 1:**
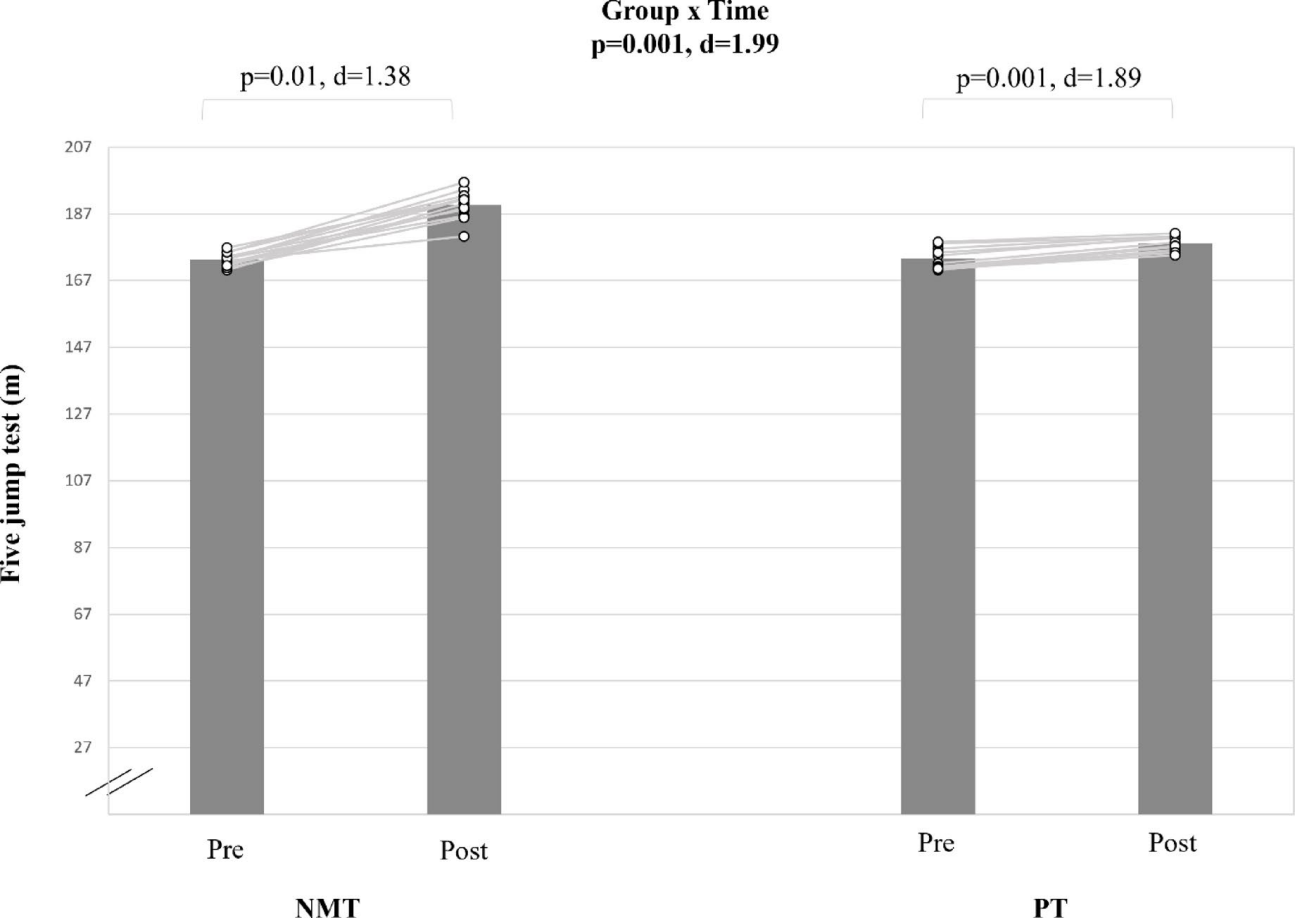
The figure displays intra-individual and group mean data illustrating the effects of two different training types on five jump test performance in pubertal soccer players. NMT: neuromuscular training; PT: plyometric training.

A large group-by-time interaction effect was found for the FJT (d = 1.99; p < 0.001) (Table 5, Fig. 1). Post hoc tests indicated significant and large pre-to-post-training improvements for PT (Δ17.3%; d = 1.89; p < 0.001). Training-induced improvements following NMT were significant but smaller in magnitude compared to PT (Δ6.0%; d = 1.38; p < 0.01) improvements.

### Change-of-direction speed

**Fig. 2:**
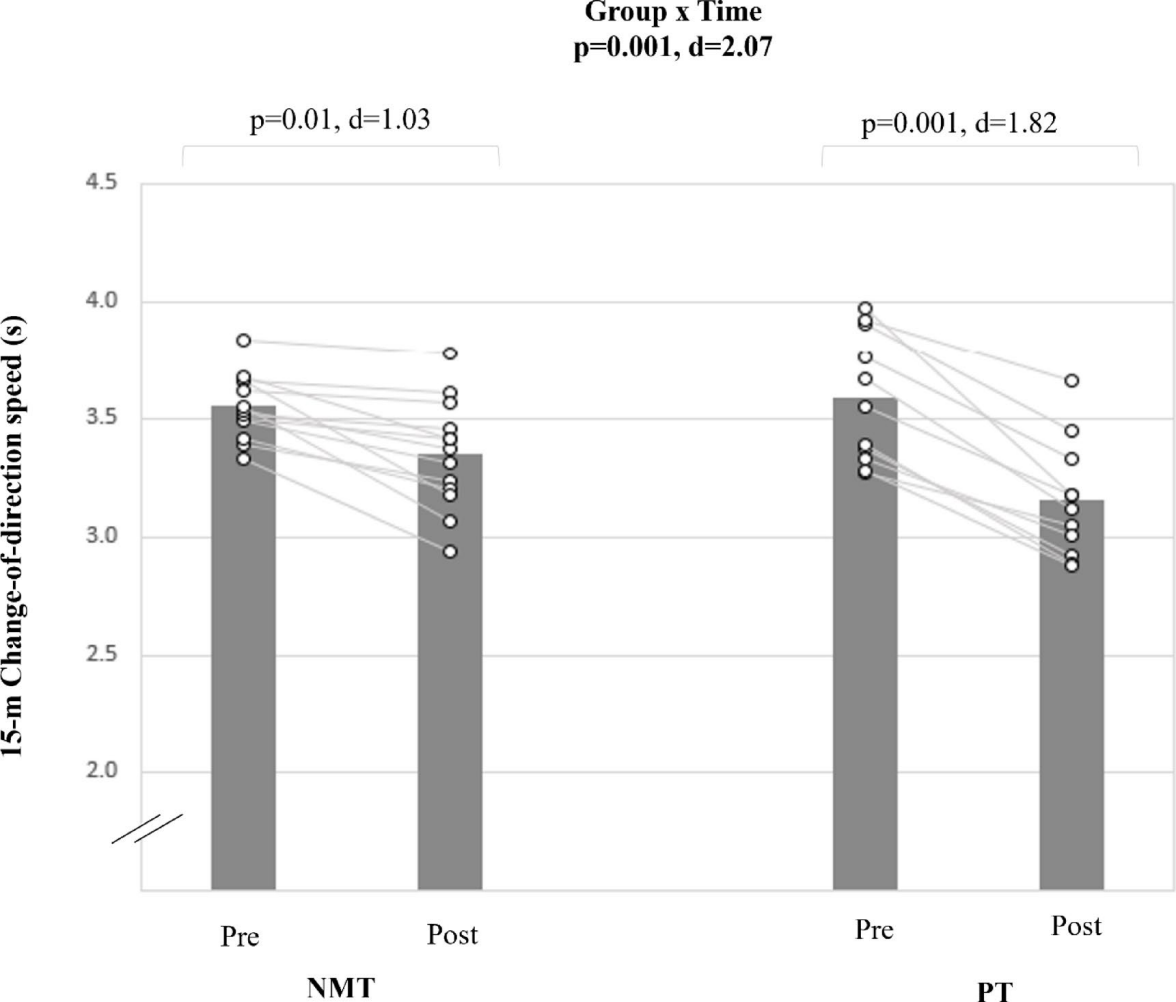
The figure displays intra-individual and group mean data illustrating the effects of two different training types on 15-m Change-of-direction speed performance in pubertal soccer players. NMT: neuromuscular training; PT: plyometric training.

A large group-by-time interaction effect was found for the 15-m CoD test (d = 2.07; p < 0.001) (Table 5, Fig. 2). PT resulted in significant and large pre-to-post-training improvements (Δ 12.1%; d = 1.82; p < 0.001). NMT produced a smaller effect size magnitude compared with PT (Δ4.5%; d = 1.03; p < 0.01).

### Linear sprint speed

**Fig. 3:**
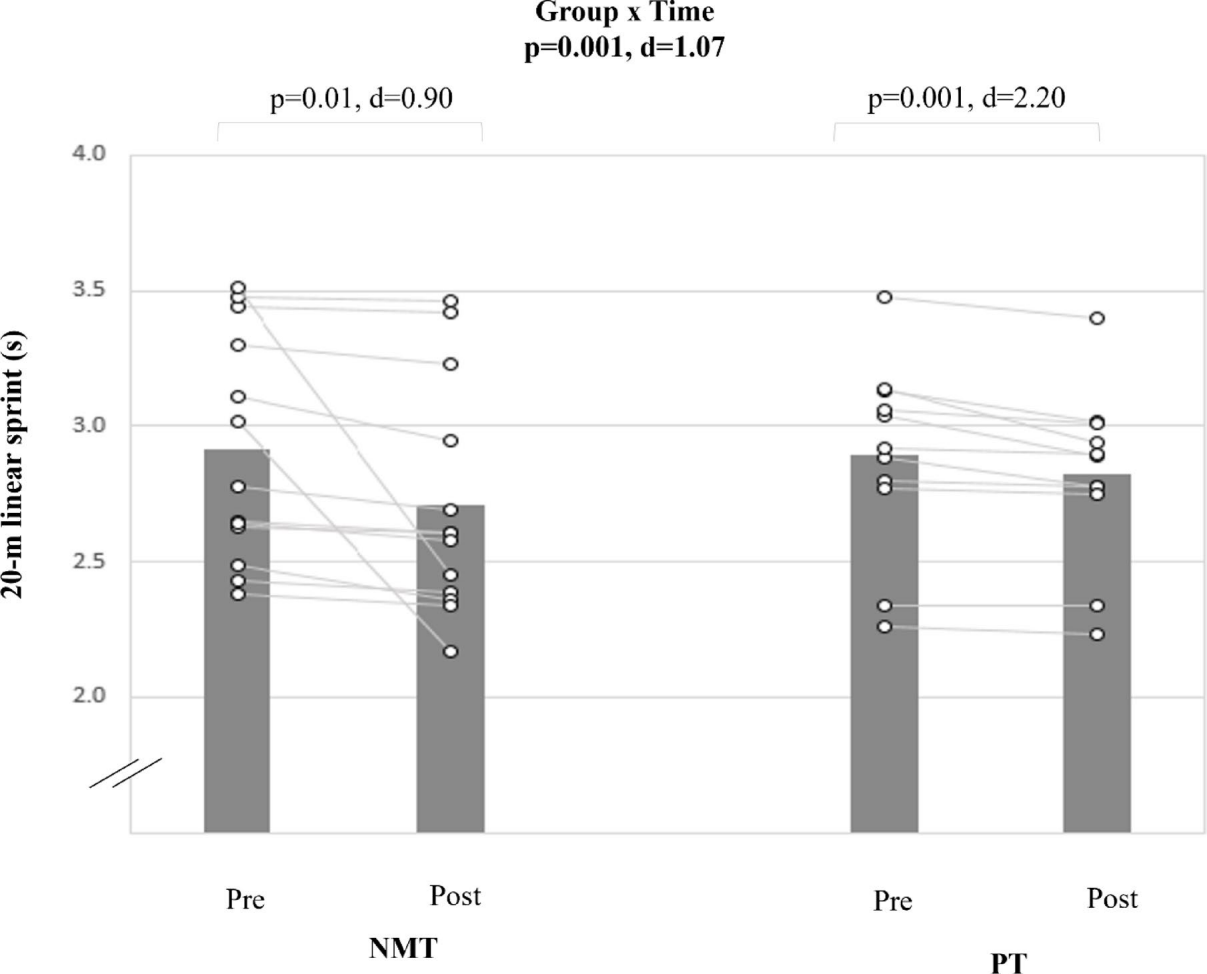
The figure displays intra-individual and group mean data illustrating the effects of two different training types on 20-m linear sprint time in pubertal soccer players. NMT: neuromuscular training; PT: plyometric training.

A large group-by-time interaction effect was found for the 20-m sprint test (d = 1.07, p < 0.001) (Table 5, Fig. 3). PT produced significant and large pre-to-post-training improvements (Δ11.1%; d = 2.20; p < 0.001). NMT resulted in moderate and significant pre-to-post-training adaptations (Δ3.2%; d = 0.90; p < 0.01).

### Mental well-being tests

#### Cognitive anxiety

**Fig. 4:**
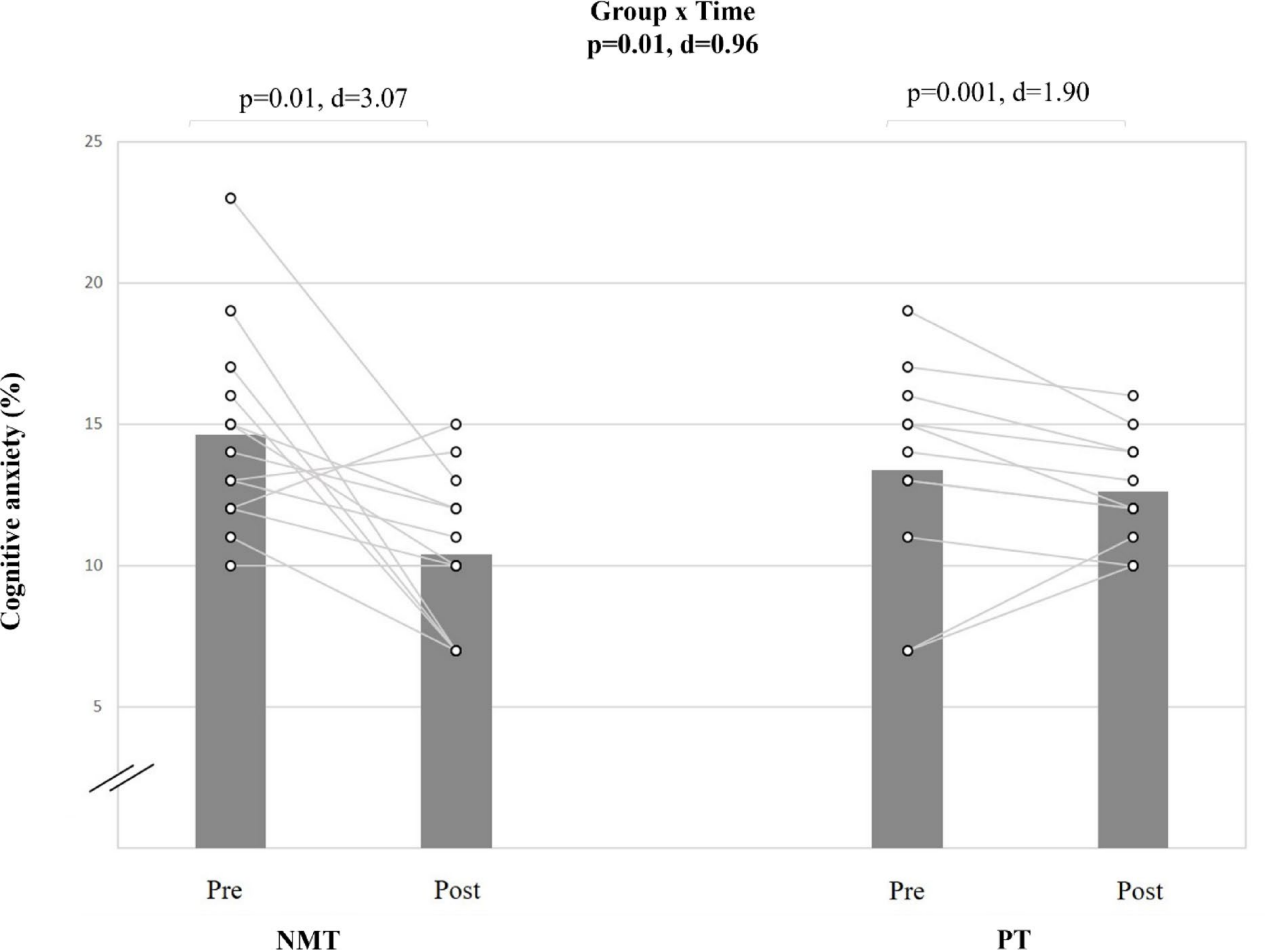
The figure displays intra-individual and group mean data illustrating the effects of two different training types on cognitive anxiety in pubertal soccer players. NMT: neuromuscular training; PT: plyometric training.

The statistical analysis showed a moderate group-by-time interaction for cognitive anxiety (d = 0.96; p < 0.05) (Table 5, Fig. 4). Post-hoc analyses revealed that NMT resulted in a very large improvement in the cognitive anxiety score (Δ28.95%; d = 3.07; p < 0.01). The PT showed significant moderate pre-to-post-training (Δ12.24%; d = 1.90; p < 0.01) improvement.

#### Somatic anxiety

**Fig. 5:**
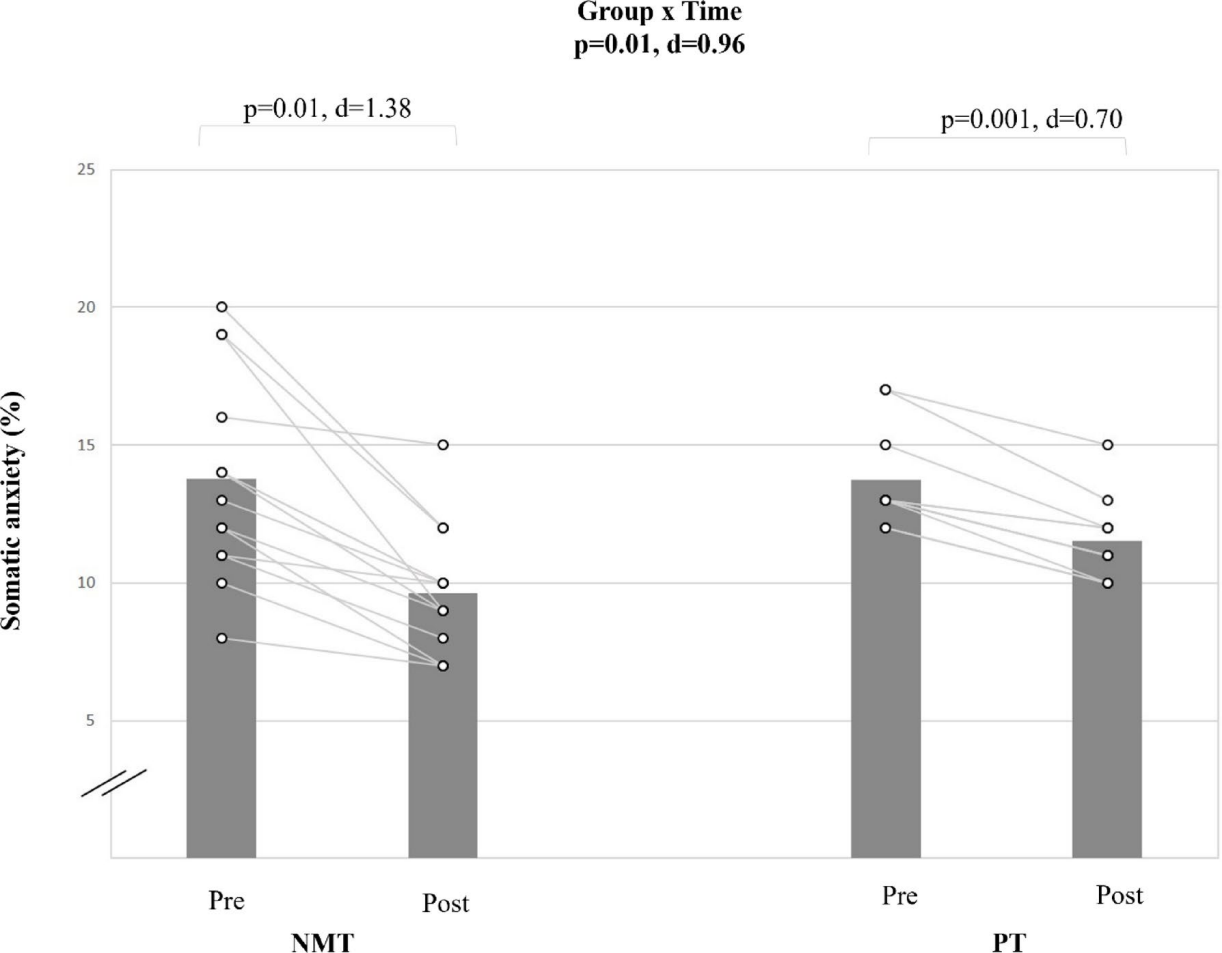
The figure displays intra-individual and group mean data illustrating the effects of two different training types on somatic anxiety in pubertal soccer players. NMT: neuromuscular training; PT: plyometric training.

A moderate group-by-time interaction was found for somatic anxiety (d = 0.96; p < 0.05) (Table 5, Fig. 5). Post-hoc analyses revealed that NMT resulted in a large somatic anxiety score improvement (Δ15.9%; d = 1.38; p < 0.001). PT caused a significant but smaller pre-to-post-training improvement (Δ8.2%; d = 0.70; p < 0.01).

#### Self-confidence

**Fig. 6:**
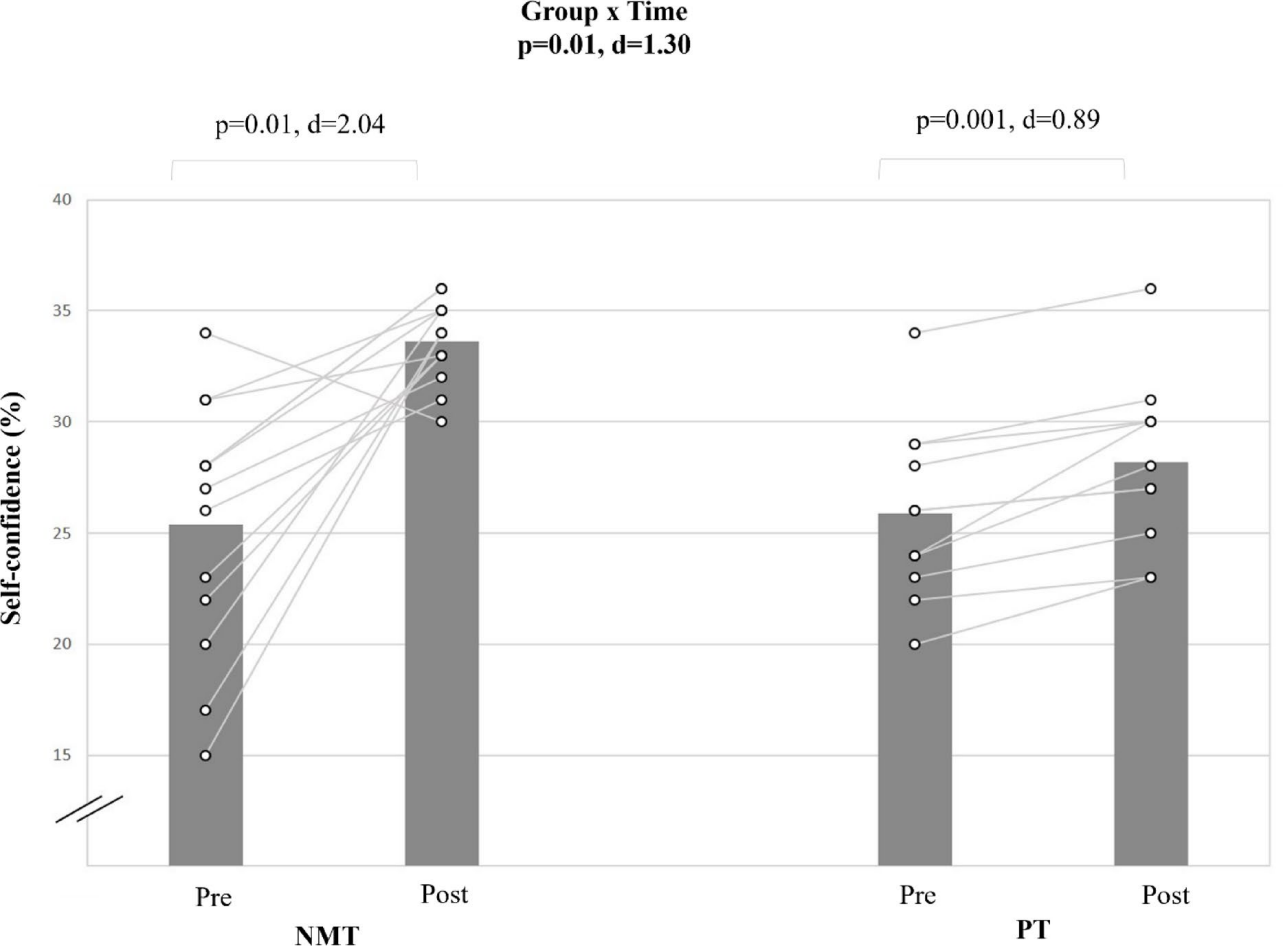
The figure displays intra-individual and group mean data illustrating the effects of two different training types on Self-confidence in pubertal soccer players. NMT: neuromuscular training; PT: plyometric training.

The analysis revealed a large group-by-time interaction for the parameter self-confidence (d = 1.30; p < 0.05) (Table 5, Fig. 6). The post-hoc analysis showed that NMT resulted in a large improvement in the self-confidence score (Δ32.4%; d = 2.04; p < 0.01). PT resulted in a significant but smaller pre-to-post-training improvement (Δ16.2%; d = 0.89; p < 0.01).

### Emotional intelligence

#### Total emotional intelligence (EC Total)

**Fig. 7:**
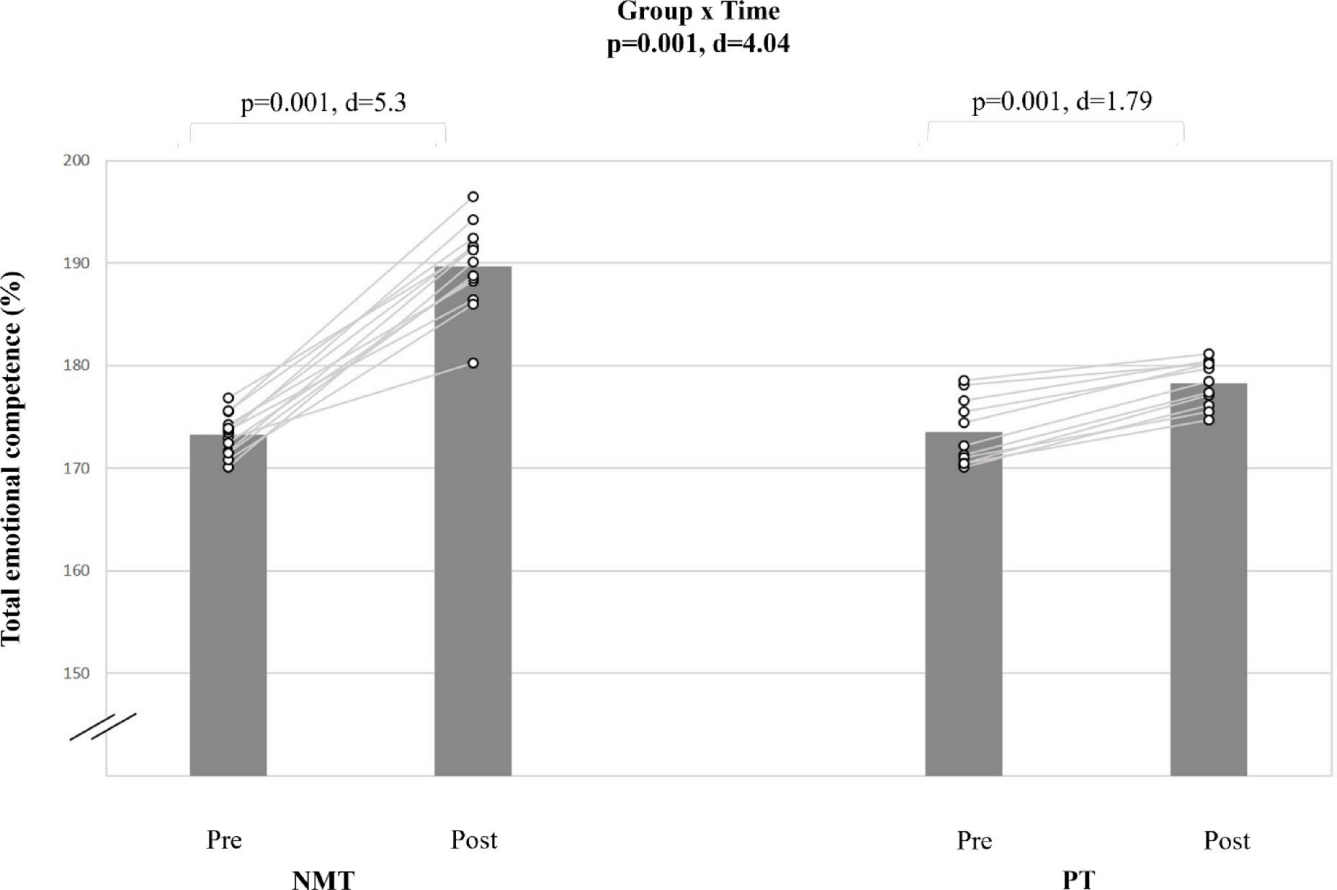
The figure displays intra-individual and group mean data illustrating the effects of two different training types on total emotional competence in pubertal soccer players. NMT: neuromuscular training; PT: plyometric training.

A very large group-by-time interaction effect was found for the EC total (d = 4.04; p < 0.001) (Table 5, Fig. 7). Post-hoc analyses revealed that NMT resulted in a larger total emotional intelligence improvement (Δ9.5%; d = 5.3; p < 0.001) compared to PT (Δ2.7%; d = 1.79; p < 0.001).

#### Intra emotional intelligence (EC intra)

**Fig. 8:**
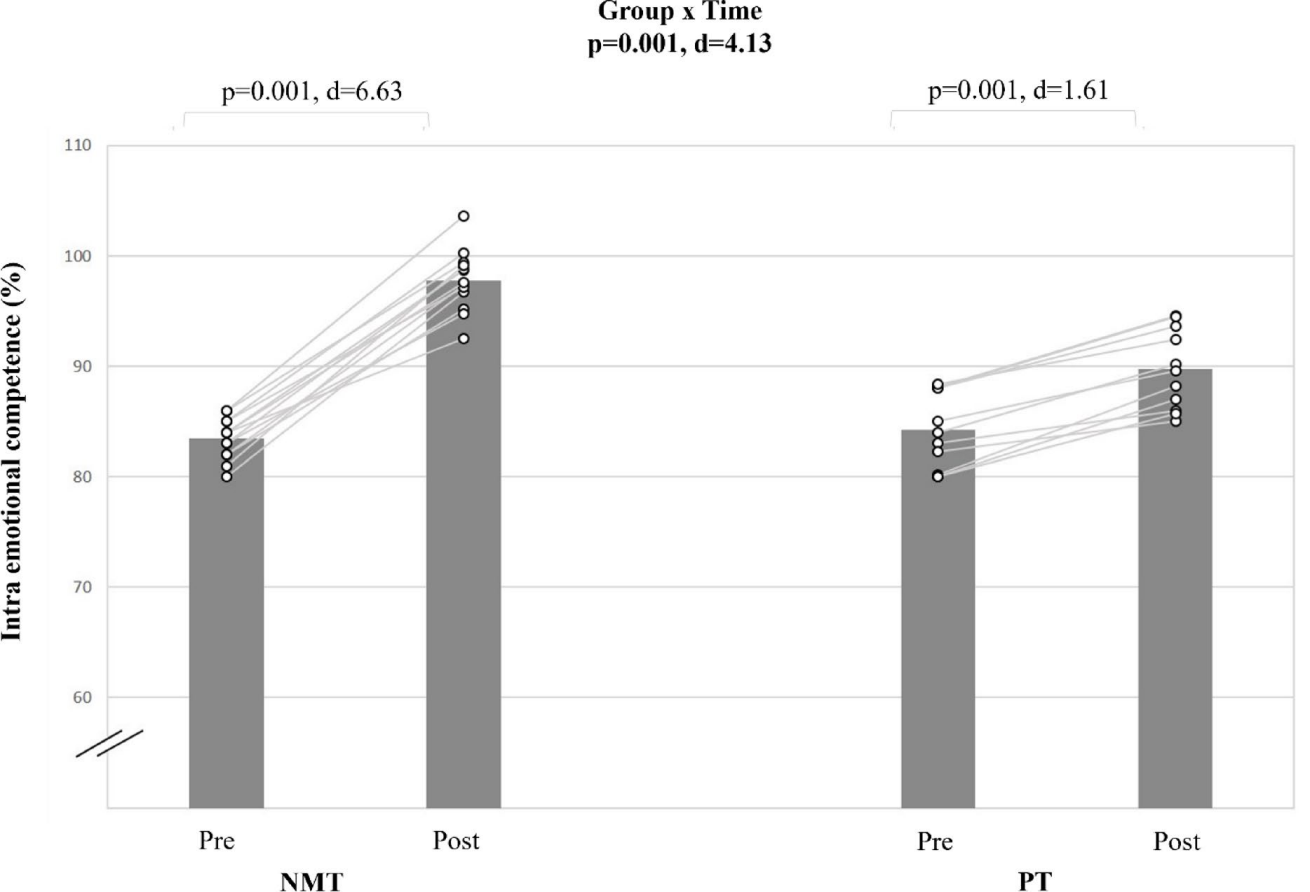
The figure displays intra-individual and group mean data illustrating the effects of two different training types on intra emotional competence in pubertal soccer players. NMT: neuromuscular training; PT: plyometric training.

A very large group-by-time interaction was found for the intra emotional intelligence score (d = 4.13; p < 0.001) (Table 5, Fig. 8). Post-hoc analyses revealed that NMT resulted in large magnitude intra emotional intelligence score improvement (Δ17.2%; d = 6.63; p < 0.001). For PT, the post-hoc analyses showed a smaller improvement in the intra emotional competence score (Δ6.4%; d = 1.61; p < 0.001).

#### Inter emotional intelligence (EC inter)

**Fig. 9:**
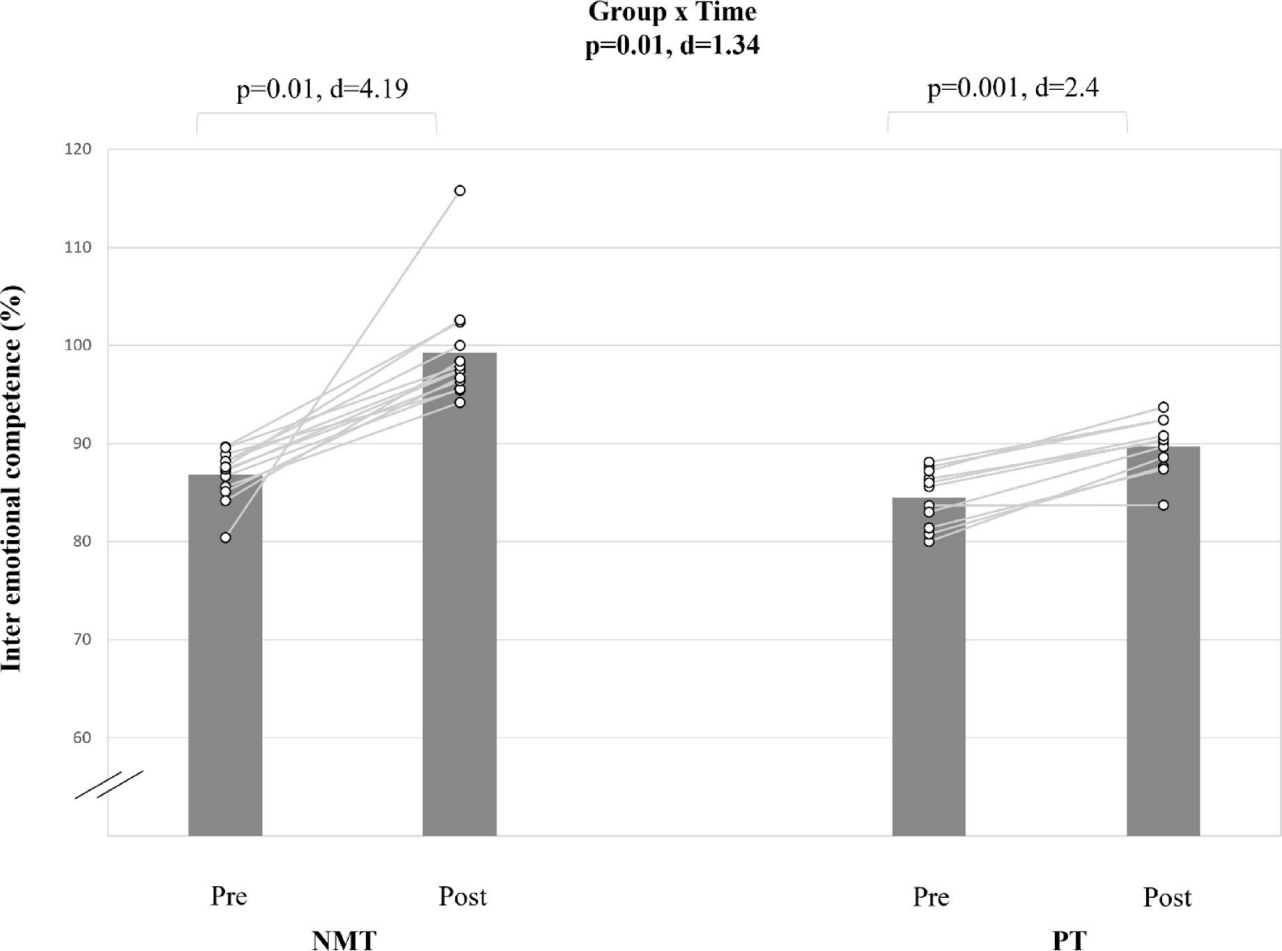
The figure displays intra-individual and group mean data illustrating the effects of two different training types on inter emotional competence in pubertal soccer players. NMT: neuromuscular training; PT: plyometric training.

The analysis showed a large interaction effect for the EC inter (d = 1.34; p < 0.01) (Table 5, Fig. 9). Post-hoc analysis indicated that NMT caused a very large EC inter score improvement (Δ7.9%; d = 4.19; p < 0.001). PT resulted in a smaller EC inter score improvement (Δ14%; d = 2.4; p < 0.001).

### Attention

**Fig. 10:**
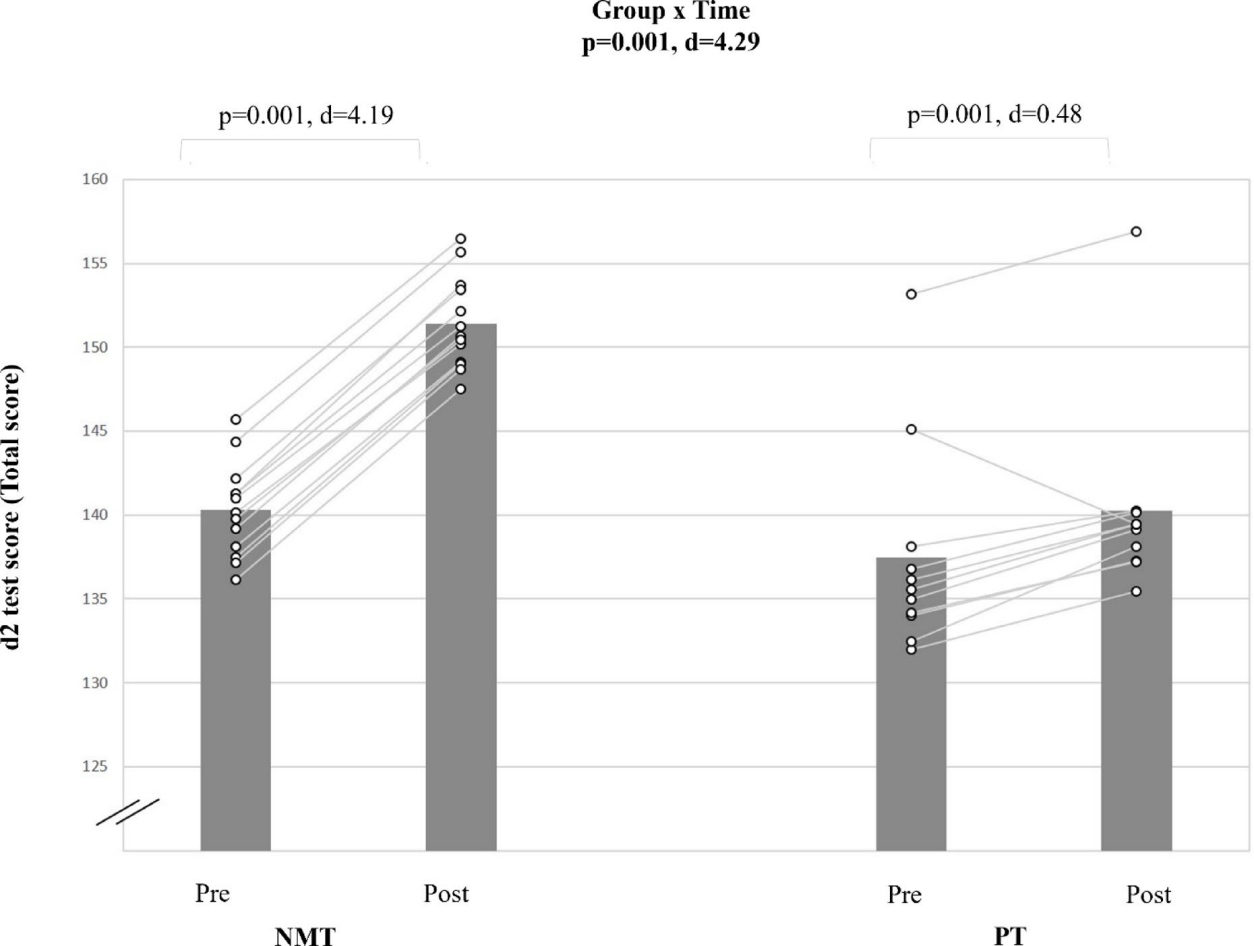
The figure displays intra-individual and group mean data illustrating the effects of two different training types on d2 test score in pubertal soccer players. NMT: neuromuscular training; PT: plyometric training. The d2 test score is derived from the number of correctly identified letters, subtracting by any error made.

A very large group-by-time interaction effect was found for the d2 test score (d = 4.29, p < 0.001) (Table 5, Fig. 10).

**Fig. 11:**
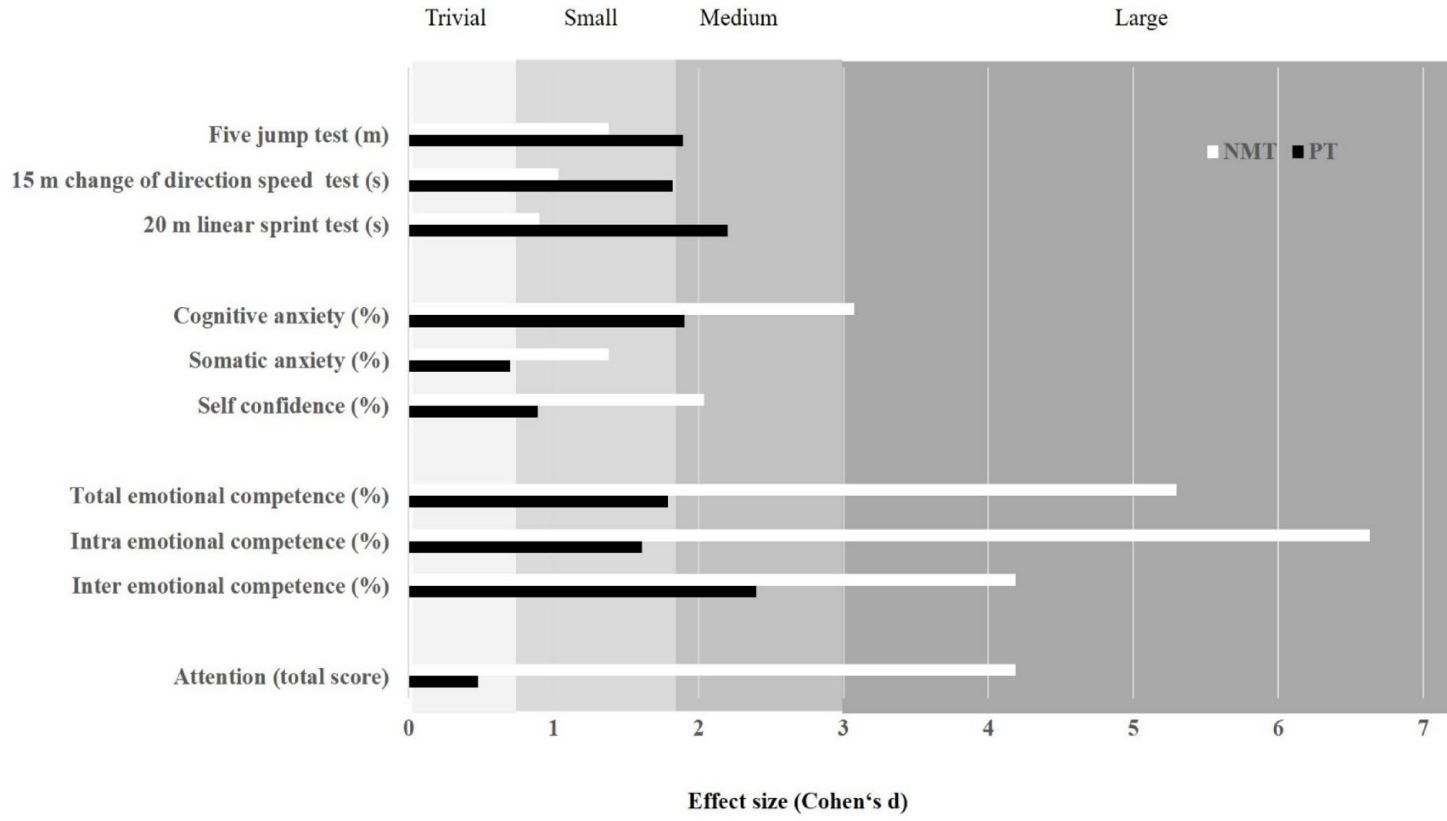
Summary of the study findings illustrated as effect sizes from group-specific pre-post changes for each measured variable based on significant group-by-time interactions.

Post-hoc analyses revealed that NMT resulted in a large improvement in d2 test score (Δ7.9%; d = 4.19; p < 0.001), while PT showed a significant but smaller pre-post improvement compared with NMT (Δ2.0%; d = 0.48; p < 0.05) (Fig. 11).

### Associations between training-induced performance changes in physical fitness and markers of mental well-being

Findings from the correlational analyses conducted on the full study sample (N = 24) are presented in Table 6. Significant and large correlations were observed between training-induced performance changes in the FJT and SA (r = 0.525, *p* < 0.001), d2 test score of attention (r = 0.643, *p* < 0.01), and EC intra (r = 0.661, *p* < 0.001). Similarly, large and statistically significant correlations were observed between changes in the 15-m CoD test and SA (r = 0.556, *p* < 0.01), attention (r = -0.594, *p* < 0.01), EC total (r = -0.656, *p* < 0.001), and EC intra (r = -0.707, *p* < 0.001). A moderate but significant correlation was found between performance changes in the 20-m sprint test and SC (r = 0.417, *p* < 0.05). No other correlations reached the level of statistical significance (all *p* > 0.05).

## Discussion

This study investigated the effects of 8 weeks of NMT versus PT, integrated into regular soccer-specific training, on physical fitness (FJT, 20-m linear sprint, and 15-m CoD speed), mental well-being (CA, SA, and SC), emotional intelligence (EC total, EC intra, EC inter), and attention (d2 test score) in male pubertal soccer players. PT produced larger improvements in measures of physical fitness, while NMT also contributed to significant gains in certain fitness parameters and elicited greater improvements in mental well-being, emotional intelligence, and attention. These findings highlight that, although both training modalities target overlapping physical fitness qualities (e.g., muscle strength, power, balance, and CoD speed), adaptations tend to reflect the primary focus of the training program. Thus, while the specificity principle^15^ partially explains the observed outcomes, the multidimensional nature of NMT indicates that its benefits extend across both physical and psychological domains, emphasizing that physiological and psychological adaptations are in accordance with the type of training stimulus applied, particularly during the pubertal developmental stage^34^. As depicted in Fig. 11, NMT and PT elicited complementary adaptations, with NMT favoring psychological outcomes and PT emphasizing physical fitness. This integrative perspective underscores the potential value of combining both training modalities in the development of youth athletes.

This study revealed greater effects of PT compared with NMT on proxies of muscle power, linear sprint, and CoD speed. PT exercises, characterized by rapid eccentric–concentric muscle actions within the SSC, effectively stimulate neuromuscular adaptations that enhance the ability to generate force rapidly^14^. Physiologically, PT targets the optimization of the stretch–shortening cycle in the muscle–tendon unit, which underlies explosive actions such as sprinting and rapid CoDs^35^. These similarities help explain the strong transfer of PT to sport-specific performance in soccer players^36^. By promoting efficient intermuscular coordination and an increased rate of force development, plyometric exercises enhance acceleration and deceleration capacities during match play^35^. Consistently, our results align with previous studies showing that PT protocols such as drop jumps and multidirectional hops lead to significant improvements in jump height, linear sprint and CoD speed, and agility performance in youth soccer players^37^.

NMT, while less reliant on the SSC than PT, provides a broader stimulus targeting balance, coordination, strength, and movement control^34^. This multidimensional approach enhances proprioception, postural stability, and intermuscular coordination, improving movement efficiency and injury resilience^38^. The cognitively demanding nature of NMT requiring attention, error correction, and adaptation to complex tasks also promotes neural adaptations linked to executive function, self-regulation, and emotional control^39^. NMT is a holistic approach targeting the physical and psychological development of pubertal athletes. By improving balance, agility speed, strength, and intermuscular coordination, NMT enhances movement efficiency and reduces injury risk^38^. Complex motor tasks require sustained attention, error monitoring, and continual sensorimotor adjustment, such demands can engage executive-control processes and have been associated with neural plasticity in networks supporting attention and cognitive control^39^. These combined physical and cognitive effects likely account for the superior gains in mental well-being, emotional intelligence, and attentional capacities observed with NMT compared with conventional PT.

Overall, NMT fosters physical fitness and psychological development by combining motor skill refinement with cognitive engagement. Its moderate intensity and challenging yet supportive structure create an optimal environment for building confidence, emotional regulation in youth^10,35,38^. While PT best develops (proxies of) muscle power, NMT serves as a holistic training modality that enhances physical fitness and mental well-being in pubertal male soccer players.

Our secondary objective was to examine whether training-induced improvements in physical fitness (via PT and NMT) were associated with concurrent changes in mental well-being. When data from both groups were pooled, significant correlations emerged between gains in physical fitness and improvements in psychological measures, supporting the findings of Hammami et al.^10^, who reported similar relationships between enhancements in jump and CoD performance and reductions in somatic anxiety, as well as increases in self-confidence among pubertal soccer players.

To explore potential links between training-induced changes in physical fitness and mental well-being, we examined correlations across the pooled sample. Significant associations were observed for selective parameters. For instance, training-induced improvements in FJT performance significantly correlated with reductions in somatic anxiety and increases in self-confidence. Changes in 15-m CoD speed were significantly associated with measures of attention and emotional competence (Table 6). The fact that not all training-induced changes in physical fitness and psychological well-being were significantly correlated, indicates that it is a rather specific and not a general phenomenon.

These observed associations likely reflect shared neurophysiological and psychobiological mechanisms. Enhanced neuromuscular function improves motor competence and body awareness, which can boost perceived self-efficacy and self-confidence, key determinants of mental well-being^40^. Physical fitness gains may also modulate the hypothalamic–pituitary–adrenal (HPA) axis, reducing cortisol reactivity and promoting emotional regulation^41^. According to Lang’s bio-informational theory, engaging and meaningful motor experiences activate interconnected physiological and cognitive-emotional systems, fostering adaptive psychophysiological responses.

Overall, improvements in physical fitness through either PT or NMT may contribute to selective psychological benefits, supporting the value of integrated physical and cognitive-emotional development in pubertal soccer players.

From an applied perspective, the complementary benefits of PT and NMT suggest that both training modalities should be incorporated during soccer training of pubertal athletes. More specifically, during the preparatory or early-season phase, greater emphasis on NMT may be advantageous to strengthen neuromuscular control, balance, and psychological readiness. As athletes progress and training loads increase, PT can be progressively introduced or intensified to optimize muscle power, strength and sport-specific performance.

Alternatively, NMT and PT can be combined within the same training week, with NMT sessions scheduled for instance during the warm-up (e.g., FIFA 11 + kids) to improve players’ readiness, high-intensity PT drills can be conducted thereafter. This integrated approach may help sustain both physical and psychological adaptations, reducing injury risk while promoting self-confidence and emotional regulation. Coaches are therefore encouraged to adopt a balanced sequencing strategy that reflects the athlete’s maturity, technical ability, and recovery capacity.

This study is not without limitations. First, the sample comprised only male pubertal soccer players, limiting the generalizability of these findings to female athletes or other age groups and sports. Future research should include diverse populations, ensuring gender balance and a wider range of developmental stages. A second limitation of the present study is the relatively small sample (N = 24), with unequal group sizes (11 vs. 13). This may have reduced statistical power and increased the risk of experiencing type II error, meaning that some effects may not have reached statistical significance due to the size of the study cohort. Hence, our findings should be interpreted with caution, and replicated in larger studies with the goal to confirm or question the observed trends. Third, correlational analyses were conducted with the pooled sample due to the limited number of participants per group. While this approach increased analytical power, it may have obscured group-specific relations and outcomes. Future studies with larger study samples are needed to confirm whether the observed associations differ between PT and NMT interventions. A further limitation is that soccer-specific agility tests were not assessed. While the study focused on fundamental physical fitness measures (proxies of muscle power, linear sprint, and CoD speed) that underpin agility, soccer-specific agility tests involve perceptual–cognitive elements, which can introduce additional variability. Future research should include soccer-specific agility assessments to better capture the combined physical and cognitive adaptations induced by NMT and PT.

In addition, the absence of a non-training control group limits the ability to determine whether the observed effects were attributable solely to the interventions or partially to regular soccer training. Moreover, the comparison was confined to NMT and PT, excluding other potentially relevant modalities such as high-intensity aerobic or isolated CoD training. Consequently, the findings should be interpreted within the context of these design constraints. Future studies should incorporate both control and alternative training groups, include additional physical fitness (e.g., muscle strength, power) and psychological (e.g., affective state) measures, and further explore how emotional intelligence develops in youth soccer players and contributes to their long-term athletic progression.

## Conclusions

This study investigated how NMT and PT, when integrated into regular soccer specific training, affect measures of physical fitness, mental well-being, emotional intelligence, and attention in male pubertal soccer players. As secondary outcome, we examined the relation between training-induced changes in physical fitness and mental well-being, emotional intelligence and attention. Our findings demonstrate that PT was significantly more effective than NMT at improving measures of physical fitness such as the FJT, the 20-m linear sprint and 15-m CoD speed tests. NMT on the other hand caused significantly larger improvements in self-confidence, emotional intelligence, and attention compared with PT.

Importantly, when we combined data from the two experimental groups, we found statistically significant and moderate correlations between training-induced changes in physical fitness and general mental health outcomes. This underscores the close connection between physical and mental development in youth athletes, highlighting the importance of addressing both aspects in holistic training approaches. For coaches, trainers, and youth development specialists, these results advocate a dual-focus training approach. First, prioritize PT when the main goal is to enhance the specific physical capacities essential for soccer performance and integrate NMT to provide crucial mental well-being, emotional intelligence and attention benefits, supporting the holistic development of the youth athlete. Thus, a balanced training model that combines both PT and NMT is recommended to optimize physical fitness outcomes, mental well-being, emotional intelligence and attention in young soccer players. Future research should investigate the long-term effects of integrated PT and NMT programs on both athletic performance and mental well-being. Exploring how these training methods can be periodized across a competitive season or adapted for different age groups and developmental stages would provide significant guidance for youth sport practitioners. Additionally, further studies are needed to examine the specific physiological mechanisms linking physical improvements to mental well-being, emotional intelligence and attention benefits, leading to a deeper understanding of how targeted training influences overall youth soccer players’ well-being.

## Data Availability

The datasets used and/or analyzed during the current study are available from the corresponding author upon reasonable request.
